# Quantitative analysis of nanoparticle transport through *in vitro* blood-brain barrier models

**DOI:** 10.1080/21688370.2016.1143545

**Published:** 2016-01-28

**Authors:** Christoffer Åberg

**Affiliations:** Groningen Biomolecular Sciences and Biotechnology Institute, University of Groningen; Groningen, The Netherlands

**Keywords:** biomolecular corona, blood-brain barrier, nanomedicine, nanoparticles, nanosafety, transwell systems

## Abstract

Nanoparticle transport through the blood-brain barrier has received much attention of late, both from the point of view of nano-enabled drug delivery, as well as due to concerns about unintended exposure of nanomaterials to humans and other organisms. *In vitro* models play a lead role in efforts to understand the extent of transport through the blood-brain barrier, but unique features of the nanoscale challenge their direct adaptation. Here we highlight some of the differences compared to molecular species when utilizing *in vitro* blood-brain barrier models for nanoparticle studies. Issues that may arise with transwell systems are discussed, together with some potential alternative methodologies. We also briefly review the biomolecular corona concept and its importance for how nanoparticles interact with the blood-brain barrier. We end with considering future directions, including indirect effects and application of shear and fluidics-technologies.

## Introduction

Nanoparticles of increasingly sophisticated variations are finding one of their most important applications as drug delivery vehicles.^1-4^ Because of their size, they may accumulate passively in tumors through the Enhanced Permeability and Retention (EPR) effect^5,6^ and thereby deliver anti-cancer drugs.^7,8^ In addition, their highly modifiable surface allows decoration with binding motifs from antibodies, proteins or small peptides, and thus a potential means for achieving selective attachment to malignant cells (active targeting). Nanoparticles are also showing promise for delivering therapeutics against diseases in the central nervous system,^9-12^ such as Alzheimer and Parkinson diseases or acquired immunodeficiency syndrome (AIDS). The central nervous system is one of the most challenging locations to reach, mainly due to the protective effect stemming from the blood-brain barrier.^12,13^ Nevertheless, nanoparticles are in general rapidly taken up by unspecialised cells,^14-16^ and using targeting moieties such as transferrin,^17,18^ apolipoprotein E,^19-23^ RVG29^24^ or Angiopeps^25^ it is hoped that a well-regulated uptake into the blood-brain barrier can be reached, and subsequently a concomitant transport across it.

On the other hand, the increased usage of nanotechnologies in consumer products have also called for consideration of whether there are any unforeseen hazards if nano-sized objects are exposed to human beings.^26-31^ Naturally, passage into the brain, and potential subsequent effects, is of particular importance in this arena.^32^ Early studies have, in fact, shown nanoparticles in the brain of rats after inhalation exposure,^33,34^ though the translocation likely occurred via the olfactory nerve,^33^ rather than across the blood-brain barrier.

Central to determining if, and *via* what mechanisms, nanoparticles pass the blood-brain barrier remain *in vitro* models. Naturally, there will always be the question of how accurate *in vitro* models represent the *in vivo* situation. Nevertheless, due to their ease of working, *in vitro* models offer distinct advantages. This is particularly so when it comes to identifying mechanisms,^35^ even if final validation will, perhaps, always have to be done *in vivo*. While *in vitro* blood-brain barrier models have been applied for a long time for the transport of molecular compounds, several, rather unique, features of the nanoscale challenge their direct adaptation to nanoparticle transport – from a quantitative, but even a qualitative, point of view. It is the purpose of this text to highlight some of the differences compared to molecular species. We start by discussing the application of transwell systems to nanoparticle transport across *in vitro* blood-brain barrier models and the many issues that may arise, particularly when it comes to quantitative measurements. Next, we propose alternative methodologies which could alleviate the issues. We continue with a brief review of the concept of the biomolecular corona, another prime difference between nanoparticles and molecular species. Finally, we end with considering potential implications and an outlook toward the future.

## Application of transwell systems to nanoparticle transport

### Transwell systems

**Figure 1.**
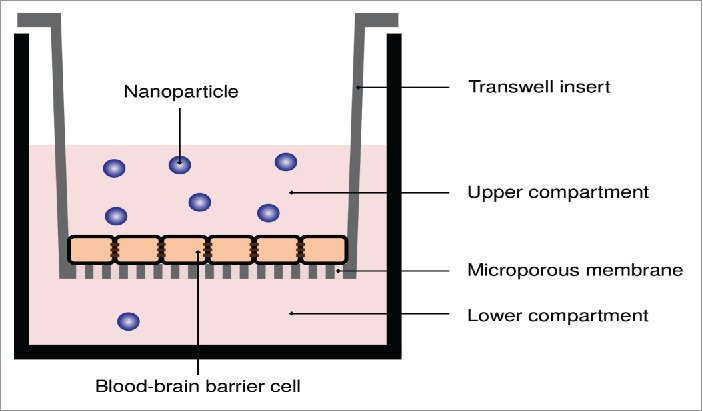
Transwell system applied to measure the transport of nanoparticles across *in vitro* blood-brain barriers. A porous membrane, upon which the *in vitro* blood-brain barrier model is grown, separates two compartments. The nanoparticles are added to the upper compartment, and the number of nanoparticles that passes through to the lower compartment is measured.

The “classical” approach to measure transport across the blood-brain barrier, or barriers in general, is by utilizing (some form of) transwell system (**Fig. 1**). Briefly, the barrier is grown on a support filter which separates 2 different compartments. The filter is porous, thus allowing transport through it, at least in principle. The choice of support filter is important, both for ensuring the formation of a good barrier and potentially to allow/minimize (depending upon application) cell migration through the filter, but will not be covered here. Once a barrier has formed, transport through the barrier of a molecule/nanoparticle is measured by replacing the solution in the upper compartment with one including the object of interest. Subsequently, the amount that has transported through to the lower compartment is measured, *e.g.*, by sampling the lower compartment and analyzing it optically, radioactively or using mass spectrometry. Based upon the amount in the lower chamber, the transport through the barrier is then quantified,^36^ *e.g.*, in terms of a permeability coefficient.

**Figure 2.**
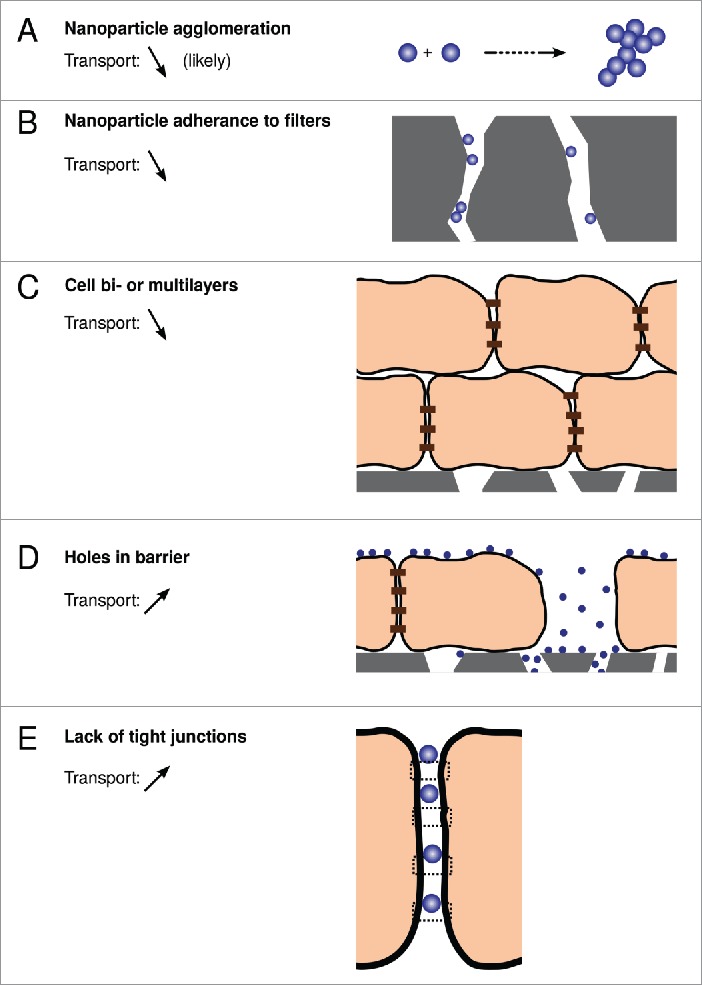
Potential issues with applying transwell systems to measure the transport of nanoparticles across *in vitro* blood-brain barriers.

This methodology has a long history and, while there may of course be technical complications in specific cases, is well-established as a general tool for measuring transport of molecular species. However, several issues arise when applying the same methodology to the transport of nanoparticles across barriers (**Fig. 2**). Some of these issues are not novel for nanoscale objects (*e.g.*, imperfections of the barriers) but would appear to be more severe for assessing transport of nanoparticles; some of the issues (*e.g.*, agglomeration) are, on the other hand, rather unique for particles.

### Nanoparticle agglomeration and adherence to filter

An issue with a distinct particle characteristics is agglomeration of the particles (**Fig. 2A**).^37^ This could potentially occur at any point along the transport: in the upper compartment, inside cells, in the filter membrane or in the lower compartment. Nanoparticle agglomeration in the upper compartment, *i.e.*, if the nanoparticles agglomerate already in the medium in which they are exposed to cells, is a serious issue. From the simplest point of view, it implies that the nanoparticles that are actually delivered to the cells are quite different from the pristine particles that the experiment set out to investigate. *E.g.*, if 50 nm nanoparticles agglomerate to form dimers, then it is these larger dimers that reach the cells, and their transport rate through the barrier may be rather different from the original particles. More importantly, agglomeration is typically uncontrolled, and it is unlikely that such a thing as a suspension of only dimers were actually to form in practice. Rather, agglomeration is more likely to result in a heterogeneous dispersion, including everything from single particles to larger agglomerates. In addition, due to the uncontrolled nature of agglomeration, the characteristics of the dispersion may be different each time it is prepared, implying a poor basis for reproducibility. Furthermore, nanoparticle concentrations used *in vitro* would typically far exceed realistic *in vivo* doses, and since agglomeration is concentration-dependent, this implies that the suspension tested *in vitro* may have little to do with the one actually used *in vivo*. In essence, nanoparticles which agglomerate in the cell medium are better not used.

Agglomeration *inside cells* is a completely different issue, because, if it takes place, it is a genuine process not having to do with idiosyncrasies of experimentation. Even if nanoparticles were to be taken up by cells individually, it is expected that they will gather in the same organelles intracellularly (*e.g.*, in sorting endosomes). Indeed, nanoparticle clusters in organelles have been observed in several different cell types,^38,39^ including in *in vitro* blood-brain barrier models.^40,41^ Whether these nanoparticle clusters are actually agglomerates (in the colloid science meaning of the word) or just several nanoparticles in the same organelle is not clear, but certainly there is the *possibility* for actual agglomeration. Agglomeration may affect the assessment of transport in several ways. The quantification of the number of nanoparticles in the lower chamber is one. For instance, if the number of nanoparticles in the lower compartment is quantified in terms of fluorescence, then the measured fluorescence has to be converted to number (concentration) of nanoparticles. However, the relation between fluorescence and number (concentration) of nanoparticles is affected by agglomeration of the particles, and there is really no adequate way of predicting the effect, nor of calibrating for it experimentally.

A different issue concerns transport through the porous support membrane. Obviously, the basic assumption behind the transwell set-up is that transport through the cell barrier is vastly slower than transport through the support membrane. However, several issues may reduce transport through the support membrane, and invalidate this assumption for nanoparticles. In severe cases, the nanoparticles may form large agglomerates as they transport through the cell barrier, and these agglomerates may be too large to pass through the pores of the support filter, especially if the particles are solid and non-deformable. The nanoparticles may also adhere to the pore walls of the filter (**Fig. 2B**),^41^ making passage more difficult for nanoparticles which do not adhere, or causing agglomeration inside the pores of the filter, which will further obstruct transport.

### Imperfections of the barrier

While *in vivo* the (healthy) blood-brain barrier forms one continuous structure, it is difficult to imagine that the same can be achieved *in vitro*. Certainly much can be done in terms of the choice of cell model, the choice of membrane support and in optimising growth conditions. Nevertheless, it is likely that there will always remain some imperfections, *on a macroscopic scale*. One example is areas where cells grow on top of each other to form bilayers, or even multilayers (**Fig. 2C**).^40^ With optimised conditions, this should not be very frequent, but may still occur. The presence of bi- or multilayers would be expected to impede transport through the cell barrier, which obviously would make the barrier appear to transport slower than it actually would *in vivo*. Nevertheless, this is a quantitative effect and would – in isolation – probably give at least the correct qualitative picture.

The presence of holes in the barrier is more severe. Holes may be rather large (**Fig. 2D**), as in areas of the barrier where cells are “simply missing,” or holes due to adjacent cells being too distant to adhere to each other, but where there is nevertheless not enough space to “fit” a full extra cell. At a more microscopic level, tight junction formation may not be completely perfect throughout the whole barrier, again resulting in holes in the barrier (**Fig. 2E**). These holes may be too small to observe using (classical) optical microscopy, where the diffraction limit sets the lower limit on what can be resolved, and difficult to find using electron microscopy, which can only investigate limited areas. The problem with holes in the barrier is that even if they are not very prevalent, they can still have a large effect.^40^ The essential complication is that transport of nanoparticles through actual cells is so slow, and transport through holes so rapid, that the contribution from transport through holes can easily mask the transport through cells (a back-of-the-envelope estimate is illustrated in ref. 40). This would – even in itself – prevent not only quantitative, but also qualitative measurements. For example, if comparing the transport of two types of nanoparticles, one could hope to subtract (or adjust for) the transport through the holes, and still gain a qualitative assessment of which type of nanoparticle exhibits the most rapid transport. However, if transport through the holes is dominating the whole transport process, then what remains after the subtraction is essentially “noise,” and cannot be used even for a qualitative estimate.

The severity of the different issues becomes worse when considered as a whole, because the different issues will affect transport in different ways: adherence to pore walls of the support filter lowers transport, while holes in the barrier will increase it. Basically, it is difficult to know, without auxiliary observations, if a measurement is over- or underestimating the actual transport.

## Methodologies for improved quantification of nanoparticle transport across *in vitro* blood-brain barrier models

Given the difficulty in using classical methods to measure – even qualitatively – nanoparticle transport through *in vitro* blood-brain barrier models, it is important to discuss alternatives. Some of the issues can be circumvented by a different choice of detection method. For example, the quantification of the number of nanoparticles in the lower compartment may be confounded due to agglomeration of the nanoparticles (inside cells or in the support membrane). This is an issue if the number of nanoparticles is quantified using fluorescence, but can be circumvented if the quantification is performed using other techniques, *e.g.*, Inductively Coupled Plasma Resonance Mass Spectrometry (ICP MS),^42^ potentially employing isotopic labeling,^43^ or radioactive labeling^44^ and detection. Furthermore, if the number of nanoparticles inside the support membrane is included in the quantification,^42^ then the adherence to filters may be less of an issue.

The transport through holes in the barrier is probably the worst complication, even if the holes are not very abundant.^40^ Qualitative information could potentially be gained using electron microscopy, which in fortunate cases can catch events of transcytosis on the basal side of the barrier.^40,42^ Naturally, this can also be used to gain qualitative information *in vivo*, as has indeed already been done.^21,45,46^ Still, the area of the barrier that can be covered using electron microscopy is limited, which precludes a quantitative estimate. Furthermore, even in the case where a nanoparticle is found at the basal membrane of an *in vitro* blood-brain barrier, inside an “evagination,” it is still possible that the nanoparticle is actually *entering*, rather than exiting, the cell – from the basal side.^40^ The nanoparticle could have arrived at the site of entry by traveling underneath the barrier, originally accessing the basal side from a hole in the barrier. This may not be apparent, because the hole in the barrier could be out of sight in the image which shows the nanoparticle entering, given the thinness of electron microscopic sectioning. Obviously, such issues are aggravated by the limited area that can be (swiftly) covered by electron microscopy. They also inhibit approaching the question of nanoparticle transport through the barrier using quantitative electron microscopy,^47^ unless the probability of finding a hole can somehow be adjusted for.

An alternative solution may be sought in live-cell imaging, as we have recently advocated.^40^ The main advantages are, first, that holes in an *in vitro* blood-brain barrier (at least those larger than the optical diffraction limit) can explicitly be looked for. Thereby, if there is a hole in one particular field of view, then this part of the barrier need not be further investigated. Second, both the transport across the barrier, and the barrier itself, can be followed – in real time. Thus, it is possible to differentiate if a nanoparticle arrives at the basal side of a cell in the barrier by traveling underneath the cells, originally from a hole in the barrier, or of it exits from a cell. Furthermore, the integrity of the barrier can be followed in time, and in this way it is possible to identify the potential formation of transient holes in the barrier. Naturally, this whole approach is only applicable to fluorescent nanoparticles, though one could imagine using it with correlative microscopy.^48^ A second disadvantage is that it demands somewhat extensive imaging work, and subsequent image analysis. This could, however, be sidestepped using high-content analysis and automated, or semi-automated, image analysis.

## The biomolecular corona and its role in nanoparticle transport across the blood-brain barrier

### The biomolecular corona

**Figure 3.**
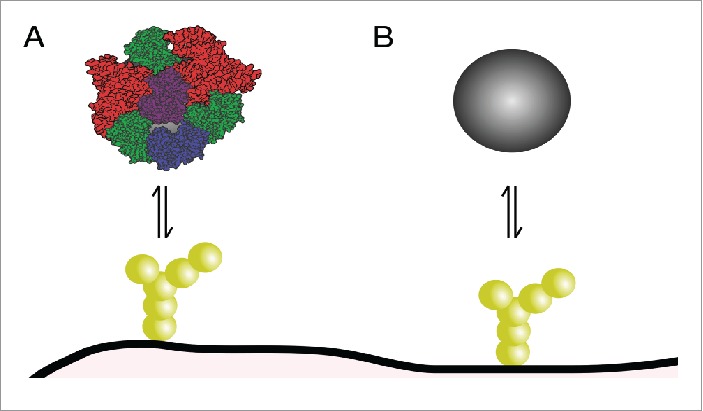
Role of biomolecular corona in nanoparticle interactions with the blood-brain barrier. (**A**) Corona-covered nanoparticle interacting with cells of the barrier *vs.* (**B**) bare nanoparticle. Only the former situation is expected to occur *in vivo*.

A different aspect – which clearly distinguishes nanoparticles from molecular species – of importance for how nanoparticles transport through the blood-brain barrier is the concept of biomolecular corona.^49,50^ The biomolecular corona refers to the adsorption of biomolecules from the environment onto the nanoparticle, forming a “corona” of biomolecules (**Fig. 3A**) that covers the original nanoparticle surface (**Fig. 3B**). The formation of such a corona is important to consider, because in any imaginable way a nanoparticle would come in contact with the blood-brain barrier *in vivo*, it would do so in the presence of a complex mixture of biomolecules in its environment, all of which could potentially adsorb. Indeed, proteins,^49,51,52^ lipids^53-56^ and sugars^57,58^ have all been found in the corona of different nanoparticles in animal-derived biological media, though the proteins are still the most studied. Furthermore, though which biomolecules adsorb differs, the same general phenomenon of a formation of corona is observed in many biological fluids,^59^ from blood serum^50,52,60,61^ to bronchoalveolar lavage fluid^56^ to urine.^59^ For nanoparticles approaching the blood-brain barrier, the corona formed in blood serum is perhaps the most important (but not the only; see below) to consider, and this is also the most well-studied.

It is a characteristic of the nanoscale, that the adsorption of biomolecules to nanoparticles can be so strong that (some of the) biomolecules will remain with the nanoparticle for much longer than it takes a cell to take up the nanoparticle.^61-64^ This is particularly so for metal,^64^ metal oxide^61,62^ and other inorganic nanoparticles.^62,63^ Still, biomolecular adsorption is sometimes also observed for nanoparticles intended as medicines, *e.g.*, those with grafted ligands, even if PEGylated to prevent unspecific adsorption.^65-68^ Effectively what it means is that, the original nanoparticle surface is not seen by the cell membrane, but rather those biomolecules which remain on the nanoparticle surface for long enough. Thus, cell membrane receptors will interact with biomolecules in the corona, and, presumably, this interaction will determine by which mechanism the nanoparticle enters the cell.^69,70^ Subsequently, one would hypothesize that once inside, the biomolecules in the corona – if, indeed, the remain associated with the nanoparticle^71-73^ – will determine to where the nanoparticle is shuttled, including if it is sent through to the other side of the barrier. This puts the spotlight on identifying which biomolecules are found in the corona, and which remain there for long times. It is noteworthy that the corona composition is not a simple reflection of the biofluid in which the nanoparticle is found. That is, if a nanoparticle is put into blood plasma with serum albumin as its main component, this does not imply that serum albumin is the main component of the nanoparticle corona.^49^ Rather, low abundant species are frequently picked up by nanoparticles, and, in general, the corona depends strongly on nanoparticle properties such as surface,^74^ size,^61,74^ shape^75^ and even concentration of the biofluid.^60^

### Importance in vivo

It seems clear that the corona will be a key determinant of how the nanoparticles are processed *in vivo*. In a sense, this has already been partly demonstrated. Thus, coating nanoparticles by polysorbate 80 results in the adsorption of apolipoproteins to the nanoparticle surface^10,76^ – in the language employed here, the formation of a corona which includes apolipoproteins – and this has been related to successful transport of a drug across the blood-brain barrier.^77^ Whether the nanoparticle transports across, or is retained in the blood-brain barrier, however remains unclear.^11^

It is important to stress that the corona is not a reflection only of the current environment, but rather exhibits history dependence.^49,78^ That is, some biomolecules may remain for very long times in the corona, while others may be exchanged if the external environment is changed. Thus, one may potentially observe very different coronae on nanoparticles reaching the blood-brain barrier *via* different exposure routes – even for the same nanoparticle. For example, if a nanoparticle is intravenously injected it may only have seen blood before reaching the blood-brain barrier, and the composition of the corona will reflect that. On the other hand, if the same nanoparticle is inhaled, it may first be exposed to lung-lining fluid, then transfer through the pulmonary barrier, before being exposed to blood and reach the blood-brain barrier.^32^ Some of the biomolecules picked up in the lung may remain, some will have been displaced upon transfer through the pulmonary barrier and others will adsorb in blood. Such effects may, ultimately, justify potential differences in how nanoparticles are processed by the blood-brain barrier depending upon the exposure route.^32^

### In vitro considerations

The corona has immense importance for *in vitro* experimentation on blood-brain barrier models. It is clear that if one wants to perfectly mimic the *in vivo* situation, then imitation of how the nanoparticle reached the blood-brain barrier is needed. In the simplest case of an intravenously injected nanoparticle, this would imply exposing the nanoparticle to an *in vitro* blood-brain barrier in the presence of blood plasma. For inhaled nanoparticles, on the other hand, this could imply mimicking how the nanoparticle interacted with lung-lining fluid, transferred through the pulmonary barrier and finally found itself in blood before reaching the blood-brain barrier. In principle this can be done, with some fidelity, by successively exposing the nanoparticle to different biofluids, for the correct period of time, before exposing it to the blood-brain barrier model. More subtle issues concern the concentration of the biofluid,^79^ and potentially species differences (including adaptation),^49,79^ *i.e.*, matching of the species origin of the cell type with the origin of the biofluid.

However, even if one does not want to perfectly mimic the *in vivo* situation, the overall presence of a corona is crucial. In the presence of a corona, nanoparticles are taken up by cells in a regulated manner, entering cells *via* energy-dependent processes^14-16^ and following endogenous sorting pathways inside cells.^14,16^ In the absence of a corona, on the other hand, nanoparticles have been observed to enter cells passively, breaking the cell membrane in the process, and subsequently diffusing around the cell cytosol.^80^ Similarly, cell death has also been associated with lack of corona.^73,80-82^ These observations may be justified in terms of the high surface energy of the nanoparticle surface.^83^ In the presence of biomolecules, the high surface energy is lowered by the adsorption of biomolecules and corona formation. Subsequent interactions with cells “occur at a lower energy scale,” and endogenous processing takes place. In the absence of biomolecules, on the other hand, the high surface energy of the original nanoparticle surface remains when in contact with cells. Thus, components from the cell membrane instead adsorbs to the nanoparticle – effectively forming a cell-derived corona^80^ – and only then is the high surface energy lowered. In essence, it is imperative to expose nanoparticles *with corona* to blood-brain barrier models, because otherwise effects that will never be seen *in vivo* may be observed. Corona formation can be ensured simply by using medium supplemented with serum (or, better yet, plasma) in the nanoparticle studies. This may not be the correct corona, because the exposure route will determine which biomolecules can be found in the corona, and ultimately those biomolecules will determine how the nanoparticle is processed by the blood-brain barrier. Nevertheless, while the detailed biomolecules will determine the specifics, it would appear that a far bigger effect results from having biomolecules there at all.

## Implications and outlook

### Indirect effects

**Figure 4.**
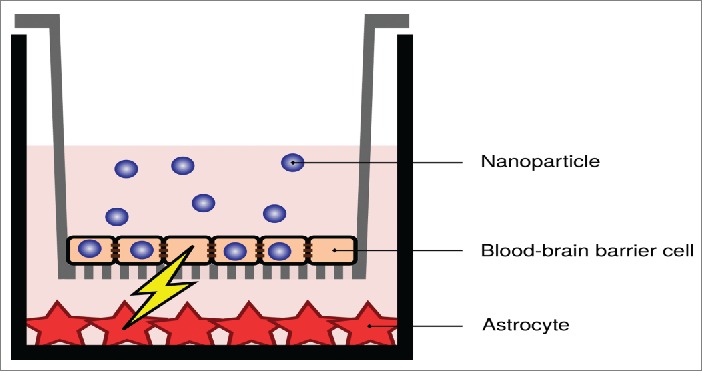
Indirect effects due to nanoparticle uptake in an *in vitro* blood-brain barrier. Despite the nanoparticles not being transported across the barrier (at least not to a significant degree), signaling takes place between the blood-brain barrier cells and astrocytic cells grown below them. Image adapted from ref. 86.

In a related arena, Case and colleagues have made the interesting observation that nanoparticles can cause signaling across cell barriers – without actually passing through the barrier.^84,85^ Such indirect effects have actually been observed also for the blood-brain barrier (**Fig. 4**), with signaling taking place between an *in vitro* blood-brain barrier and astrocytes grown below it.^86^ It is obviously imperative that studies on indirect effects are carried out with many of the issues discussed here in mind. For instance, if *in vivo* a nanoparticle is able to exert an indirect effect across the blood-brain barrier, but *in vitro* crosses an imperfect blood-brain barrier through holes in the barrier, what is actually an indirect effect could be misinterpreted as a direct effect. Obviously the opposite could also occur. One could imagine even more complicated scenarios, where *in vivo* signaling takes place but not to a significant extent, whereas the nanoparticle passes through holes in an imperfect barrier, picks up the signaling molecule on the other side of the barrier through adsorption, and subsequently delivers it to the receiving cells, at a higher dose. Such variations on the “trojan horse” effect^87^ could be a significant challenge to dissect, if imperfections in the barrier are not considered.

### Application of shear stress

One of the more important elements missing from *in vitro* blood-brain barrier models, at least in their present typical incarnation, is shear stress on the cells. This may be an area where more attention is needed in future, for two reasons: First, application of shear stress may improve the quality of *in vitro* blood-brain barriers. Thus, it has been shown that shear stress changes the expression of a large number of genes in endothelial cells,^88,89^ and also that tight junction formation is promoted in an *in vitro* blood-brain barrier model.^90^ Such observations suggest that more well-defined *in vitro* blood-brain barrier models may be achievable by applying shear stress. Conversely, it is imperative to ensure that imperfections are not introduced by applying shear stress, *e.g.*, due to the flow “washing away” cells, thus leaving the barrier with holes in it. Second, the adaptation to shear stress could also directly affect how cells of the blood-brain barrier take up and subsequently transport the nanoparticle. Indeed, nanoparticle uptake into barriers has been shown to be affected by application of shear in *in vitro* systems,^91-93^ and there are also theoretical arguments to support such reports.^94,95^

### Fluidics and miniaturization

The general adaptation of microfluidics as a methodology^96^ could prove to be a significant factor in advancing knowledge on nanoparticle transport across the blood-brain barrier. Due to the smaller areas involved, it may be possible to form much improved barriers, without holes and other imperfections. Coupled to the possibility of continuous, and well-defined, shear stress, this could give much refined *in vitro* “brain-on-a-chip” systems. Looking further into the future, it is conceivable that researchers could move beyond even that. Above it was discussed how the nanoparticle biomolecular corona potentially could depend upon the exposure route, exemplified by an intravenously injected nanoparticle or one reaching the blood-brain barrier through inhalation. The latter process may be possible to mimic – a “human-body-on-a-chip” approach^97^ – using coupled fluid reservoirs representing the different environments and letting the nanoparticles pass these reservoirs in succession before finally arriving to an *in vitro* blood-brain barrier model.

### Following nanoparticles through the barrier, in detail

Whether for improving therapeutic delivery of nanomedicines against neurodegenerative disorders, or whether out of concern for eventual hazards posed by nanoparticles passing into the brain, vital for the future will be to understand what nanoparticle properties enable efficient uptake into and transport across the blood-brain barrier. Knowing the most important properties will enable engineering the nanoparticles so as to avoid unwanted accumulation (in the latter case) or optimise desired accumulation into the brain (in the former). Likely *in vitro* blood-brain barrier systems will play a lead role in this effort, because they enable a much more rapid, economical and ethical screening of nanoparticle properties than does *in vivo* experimentation. Ultimately, it may be necessary to dissect the full transport pathway through the cells, from the early endocytic events, *via* the sorting stage and to the eventual transcytic event. If no simple correlation between nanoparticle properties (including their biomolecular coronae) and transport efficiency can be found, then this is likely the only option available in order to understand what facilitates, or impedes, efficient transport through the barrier. *In vitro* models “will be key in this endeavor” because they allow observing each event as it happens – live – and thereby to build knowledge of each step encountered by nanoparticles on their way through the blood-brain barrier.
